# Overly optimistic picture of current state of cross-border patient care in ‘Going the extra mile’ study

**DOI:** 10.1136/bmjqs-2020-011146

**Published:** 2020-03-27

**Authors:** Dionne S Kringos, Femke Jansen, Tessa Jansen

**Affiliations:** Public Health, Amsterdam UMC, University of Amsterdam, Amsterdam Public Health research institute, Amsterdam, The Netherlands

**Keywords:** health services research, health policy, hand-off

As international health services researchers, we read the paper by Beuken *et al* 1 with great interest. However, in our opinion the authors are too optimistic about the actual volume of current cross-border healthcare. The study also fails to address the role of healthcare professionals to support patients in *their* cross-border experience, and ultimately, proposes far too modest directions for action. Consequently, it misses the opportunity to go the ‘extra mile’ needed for this important topic.

In our opinion, the authors are idealistic in writing that a relatively large number of Europeans are receiving cross-border healthcare. This lacks important nuances. Five years after the adoption of Directive 2011/24/EU on the application of patients’ rights in cross-border healthcare, still, less than 0.05% of European Union (EU) citizens receive healthcare treatments abroad under the Directive.^2^ Most cross-border healthcare claims are made within the context of the Regulation No 883/2004 on the coordination of social security systems. This amounts to approximately 2 million claims a year for unplanned treatments abroad. There is ample research showing citizens’ lack of awareness about the Directive itself and the existence of National Contact Points, explaining the scarcity of patients seeking care abroad under its terms.^3^

Moreover, we were surprised by the fact that the healthcare professionals participating in the study of Beuken *et al* 1 showed little awareness of the burden current cross-border healthcare arrangements put on patients. Examples of frequently reported barriers relate to the use of prior authorisation, administrative requirements and reimbursement systems.^4 5^ To ease the handover of patients, the focus of providers should not solely involve their personal contacts with colleagues and alignment issues. Their attention to the patients’ perspective is of paramount importance.

The study very importantly identifies healthcare professionals’ perceived lack of control in cross-border patient handover. Issues such as information transfer, differences in task division and education, use of tools and protocols and cultural and language differences are complex matters. Even at the national level, some of these issues are difficult to solve across healthcare institutions and healthcare providers, let alone across borders and healthcare systems.

The authors provide solutions in the direction of peer discussions among collaborating healthcare professionals to increase mutual awareness and understanding of differences in expectations and approaches. While this may contribute to the quality of relationships between collaborators it is not likely to change current practices and may not sufficiently contribute to (or even worsen) a provider’s perceived empowerment. The authors should have considered recommendations geared towards actors that actually have a mandate to change current cross-border arrangements, such as National and Regional Contact Points, the Network of Regional Hubs, the European Committee of the Regions and the EU. Moreover, the authors seem to be unaware of the recent public consultation launched (November 2019 to January 2020) by the European Committee of the Regions through its Network of Regional Hubs, to investigate the implementation of the Directive at the territorial level. We hope the participating healthcare professionals of Beuken *et al*’s study have had the opportunity to also contribute to this consultation. Perhaps the results of this consultation will provide a clearer direction for overcoming existing challenges to cooperation among border regions and healthcare professionals.
